# Unveiling myometrial SP/Krüppel-like factors as drivers of parturition: insights from transcriptional and endocrine regulatory networks

**DOI:** 10.1530/EC-25-0296

**Published:** 2025-09-02

**Authors:** Frank A Simmen, Rosalia C M Simmen

**Affiliations:** Department of Physiology & Cell Biology, University of Arkansas for Medical Sciences, Little Rock, Arkansas, USA

**Keywords:** specificity protein, Krüppel-like factor, myometrium, parturition, endocrine, labor onset

## Abstract

**Graphical abstract:**

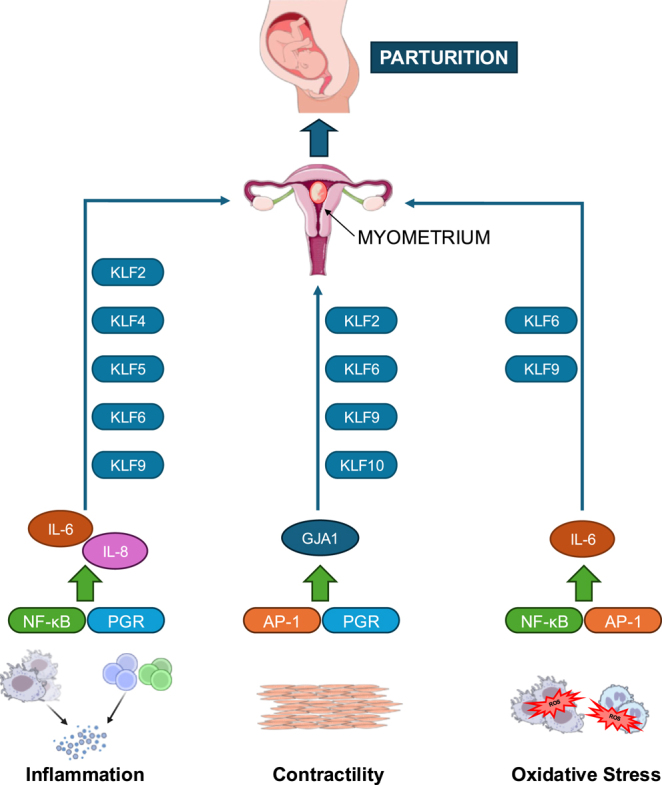

**Abstract:**

Parturition is a complex process that is subject to multifactorial regulation by signals originating from the fetus, placenta, and uterus. The uterine myometrium is a central player for achieving successful pregnancy to term, requiring its transformation from a quiescent and growing entity to one of significant contractility within a narrow window of intricately regulated events. Receptors for the steroid hormones progesterone and estrogen are critical to myometrial transformative processes. Specificity protein/Krüppel-like factor (SP/KLF) family members are transcriptional regulators that play key roles in human physiology and pathology, in part as members of steroid hormone signaling networks. Here we review findings to establish the molecular rationale for a novel function of multiple SP/KLF proteins in human parturition. Support for their potential crucial roles includes: i) differential levels of myometrial expression at labor; ii) transcriptional regulation of myometrial expressed genes; iii) functional abilities to mechanistically influence steroid hormone signaling; and iv) parturition-associated hormonal control of their expression. Understanding myometrial SP/KLF networks in the physiologic control of myometrial contractility may have therapeutic relevance for management of aberrant labor onset to reduce maternal and neonatal morbidity and mortality.

## Introduction

Parturition is a complex process that is under multifactorial regulation by signals largely originating from the fetus, placenta, and uterus (1). During the transition to labor, the human uterine myometrium transforms from a relatively quiescent, non-contractile, expandable state to one of high contractility, culminating in coordinated and heightened activity to enable fetal birth. Myometrial (smooth muscle) cells, vascular endothelial and vascular smooth muscle cells, and uterine immune cells are functionally associated with the process of attaining myometrial contractility at term (2). Subtle, though significant, changes in the cellular composition, numbers, and phenotypes of these cell types, and their altered transcriptional programs under the primary influence of the pregnancy-associated steroid hormones progesterone (P_4_) and estrogen (E_2_), acting via their cognate receptors, underpin these dynamic events in the myometrium (2, 3). Accompanying myometrial tissue remodeling near term is a major transition in immune cell landscape, mostly occurring locally in the myometrium and at the fetal–maternal interface, and also in the maternal circulation (2, 4, 5). The process of labor is initiated when strong uterine contractions, cervical ripening, and rupture of fetal membranes, exquisitely coordinated via intracellular, intercellular, and inter-tissue communication, occur in synchrony (1, 3).

Preterm labor, defined as labor that starts before 37 weeks of pregnancy and typically leads to premature birth, is a significant global cause of neonatal morbidity and mortality (3, 5). One long-standing hypothesis is that preterm labor represents the early (premature) activation of myometrial pathways and associated events that occur during normal labor (6). A disorder at the other end of the parturition spectrum is prolonged labor, defined as labor extending beyond 20 h for nulliparous women and 14 h for multiparous women. Prolonged labor also poses significant risks to both maternal and neonatal health due to its associated complications of fetal macrosomia, stillbirth, peripartum infections, C-section necessitating labor induction, and postpartum hemorrhage (5, 7). The absence of effective clinical options for preterm labor, and equally for delayed/prolonged labor, warrants further understanding of key molecular processes and regulatory pathways underpinning normal labor to mitigate the adverse consequences of abnormal onset.

Large-scale alterations in myometrial chromatin accessibility and transcription factor binding to gene promoter and regulatory regions, and as a consequence, in myometrial transcriptomes, occur during the transition from myometrial quiescence to intense contractility and labor onset (1, 2, 3, 4, 5, 6). Current state-of-the-art technologies of chromatin immunoprecipitation (ChIP) coupled with DNA sequencing (ChIP-seq) and RNA sequencing (RNA-seq) have revealed many details of chromatin changes and transcriptomic changes in the myometrium during this critical window. However, because these studies have mainly focused on a few well-known transcriptional regulators in the uterus (discussed briefly below), the identities of potentially consequential new players remain a significant knowledge gap. Moreover, since dynamic changes in local and peripheral immune signatures occur correspondingly with heightened myometrial activity at term (4, 5, 7), transcriptional mechanisms underlying the integration of inflammation and myometrial contractions merit further inquiry.

## Known myometrial transcriptional regulators of labor onset

Many excellent reviews have focused on this subject, and readers are directed to these published studies for further details (1, 3, 6, 8). Activator protein 1 (AP-1), nuclear factor kappa B (NFκB), estrogen receptor-α (ESR1), and progesterone receptor (PGR) isoforms A and B (PGR-A, PGR-B), each with well-defined signaling pathways, constitute nodal regulators of the myometrial transcriptome during the transition to labor. Briefly, myometrial contractile quiescence during pregnancy and before parturition is maintained largely by higher circulating levels of progesterone (P_4_) relative to estrogen (E_2_). PGR-A can repress PGR-B transcriptional activity in myometrial cells (9), and an increase in the relative ratio of PGR-A to PGR-B in the myometrium at term (considered a state of P_4_ resistance) leads to activation of expression of multiple contractility-associated protein (CAP)-encoding genes (1, 3, 6, 10, 11). Alterations in the uterine expression of P_4_-metabolizing enzymes, leading to decreased albeit not total withdrawal of P_4_ levels, and changes in functional interactions of PGRs with receptor co-activators and co-repressors, and with other transcriptional regulators such as NFκB and AP-1 (3, 12, 13), also contribute to withdrawal of P_4_/PGR actions. By contrast to PGR signaling, which declines in the myometrium at term, E_2_/ESR1 signaling exhibits wide-ranging functions in this tissue throughout pregnancy. Preterm, it drives myometrial hyperplastic and hypertrophic growth and stimulates CAP gene expression, while at term, it attenuates PGR function and promotes pro-inflammatory signaling to attain a highly contractile myometrial state for labor onset (14, 15). Specific miRNAs in the human myometrium are reported to be differentially expressed during the transition to labor (3, 16). Since these miRNAs can target mRNAs for ESR1, PGR isoforms, NFκB, and specific immune and cytokine pathway genes (3, 14, 16), their regulated expression, while yet to be mechanistically defined, is essential for appropriate responses to parturition signals.

Recent studies have identified additional transcriptional regulators based on their myometrial expression and presumed functions during late pregnancy and parturition. These nuclear proteins include Forkhead Box O1 (FOXO1) and FOXO3 (17), interferon regulatory factors (IRF1 and IRF5) (18), several HOX proteins (19), MAFF (20), MYB and ELF3 (21), C/EBPδ (22), and CNOT1 (23). While many of these proteins are known to mediate pro-inflammatory, immune, and PGR signaling pathways, an integrated picture of whether, how, and when they work together to program myometrial phenotype and hence parturition remains to be defined.

## SP/KLFs as novel regulators of labor onset

### SP/KLF family members

The human genome contains nine distinct yet related SP (specificity protein) genes (designated SP1–SP9) and seventeen KLF genes (designated KLF1-KLF17; KLF18 is likely a pseudogene in humans), together comprising the conserved SP/KLF family. Members of this family are characterized by the presence of Cys(2)/His(2) zinc finger motifs in their carboxy-terminal domains that confer preferential binding to GC/GT-rich sequences in gene promoter, enhancer, and super-enhancer regions of chromosomal genes. Their importance in mediating transcriptional events and dynamic regulatory networks was established early on from studies that demonstrated their ability to interact with each other and with other transcriptional regulators at the level of specific target genes, and to recruit histone deacetylases (HDACs) and co-repressor mSIN3A to transcriptional complexes in chromatin (24). Because of their ubiquitous and generally overlapping presence in tissues and cells, their functions in mediating growth, differentiation, apoptosis, and development are wide-ranging and may be additive, compensatory, cooperative, or circuitous (24). Several reviews have provided comprehensive narratives of the SP/KLF family of proteins in female reproduction, specifically in relation to their abilities to regulate steroid hormone responses (24, 25). However, only a few studies have examined these proteins in myometrial functioning during pregnancy and at term. An early report showed that expression of the human myometrial connexin 43 gene (an important CAP-encoding gene; GJA1) is dependent on SP1 binding to two SP1 *cis* elements (GC boxes) located in its proximal promoter region (26). Other reports have demonstrated binding of SP1, SP3, and SP4 to promoter or enhancer elements of several myometrial expressed genes (27). Furthermore, SP1 has been reported to affect PGR-B transactivity in uterine endometrium (28) and by analogy may have similar role(s) in the myometrium. In the following sections, the molecular rationale for novel roles of multiple SP/KLF proteins in human parturition is described. Findings supporting their potential critical functions at term include their differential levels of myometrial expression at labor, their transcriptional regulation of myometrial expressed genes, their functional abilities to mechanistically influence steroid hormone signaling, and the parturition-associated hormonal control of their expression.

### Myometrial expression of SP/KLFs at labor

**Figure 1:**
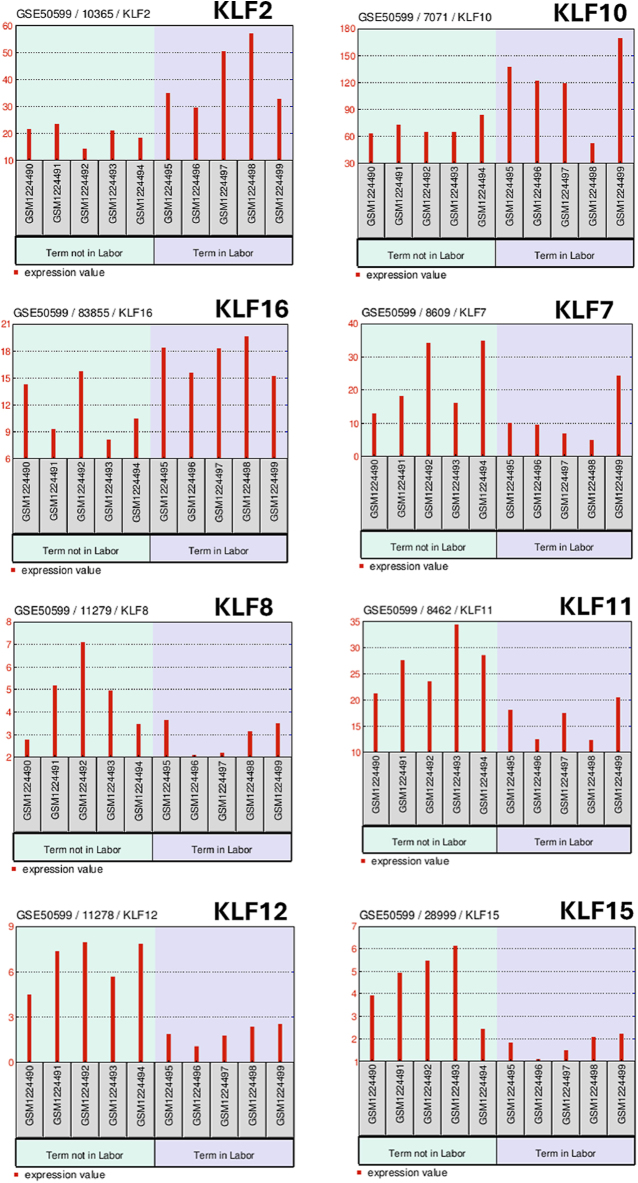
Myometrial KLF transcripts that differed in abundance between women at term/not in labor and women at term/in labor. Shown are transcript counts (y-axis) in RNA-seq data for the indicated KLF genes in myometrial samples from five individual women at term/not in labor (green shading) and five women at term/in labor (violet shading) (61). The RNA-seq data (NCBI, GEO GSE50599; https://www.ncbi.nlm.nih.gov/geo/query/acc.cgi?acc=GSE50599) were accessed and analyzed using GEO2R (https://www.ncbi.nlm.nih.gov/geo/geo2r/) (March 26, 2025). Results were evaluated using the Benjamini & Hochberg (false discovery rate) test with a significance level cut-off of 0.05. The order of most significant differences between ‘not in labor’ and ‘in labor’ groups (based on *P* value) was KLF12 > KLF2 > KLF15 > KLF11 > KLF10 > KLF7 > KLF16 > KLF8. SP1, SP2, SP3, and SP4 transcript levels did not differ between groups; SP5–9 transcripts were very low to undetectable. Elevated myometrial KLF2, KLF10 and KLF16 transcript levels (2.2-fold, 1.7-fold, 1.6-fold, respectively) were characteristic of women in labor; conversely, lower myometrial KLF7, KLF8, KLF11, KLF12, and KLF15 transcript levels (0.49-, 0.66-, 0.61-, 0.31-, and 0.41-fold, respectively) were evident in myometrium from women in labor.

Figure 1 summarizes our analysis of an RNA-seq database (NCBI, GEO GSE50599; https://www.ncbi.nlm.nih.gov/geo/query/acc.cgi?acc=GSE50599) for possible differential expression of KLF and SP gene transcripts in whole myometrium of women at term/not in labor vs women at term/in labor. In myometrium of term women in labor, transcript counts for KLF2 (2.2-fold), KLF10 (1.7-fold), and KLF16 (1.6-fold) genes were higher, while those for KLF7 (0.49-fold), KLF8 (0.66-fold), KLF11 (0.61-fold), KLF12 (0.31-fold), and KLF15 (0.41-fold) were lower relative to myometrium of term women not in labor (Fig. 1). No significant differences in expression between these two groups were noted for the other KLFs. Analysis of transcript counts for myometrial SP genes showed no significant differences in SP1–SP4 levels and very low or absence of detectable transcripts for SP5–SP9 in term women regardless of labor status. The major shifts in gene expression for a subset of KLFs during myometrial transition to contractility implicate KLF family members in the transcriptional regulation of key myometrial genes for labor onset. Still, specific myometrial gene targets of these differentially expressed KLFs have yet to be defined. Previous studies have shown that the trans-repressor functions of KLFs typically dominate over their trans-activator actions (29). The substantial increase in gene expression for KLF2, KLF10, and KLF16, coupled with the corresponding modest decrease in expression for KLF7, KLF8, KLF11, KLF12, and KLF15 genes at term with accompanying labor vs non-labor, suggests global activation as well as repression of diverse downstream gene repertoires to support the acquisition of a myometrial contractile phenotype leading to parturition.

**Figure 2:**
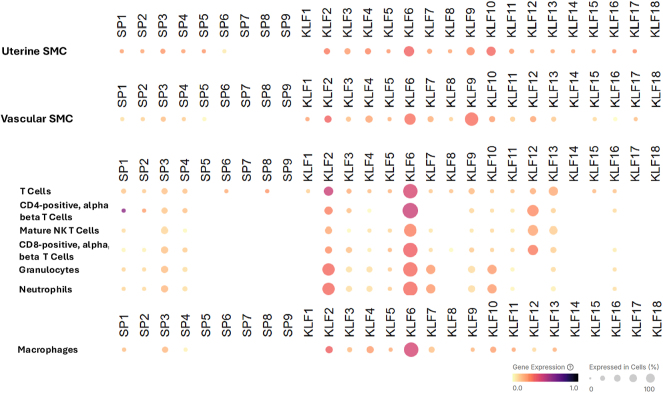
Relative RNA expression of SP/KLF genes in smooth muscle cell (SMC) and other cell types of the human uterus. Single-cell RNA sequencing (scRNA-seq) data (Tabula Sapiens consortium) for human uterus were accessed (beginning on March 28, 2025) using the CZ CELLxGENE: discover tool and corresponding data portal (https://cellxgene.cziscience.com/) (62, 63). Shown are the dot plots obtained after querying human uterus scRNA-seq data (from two individuals, ages 38 and 45 years) for all SP and KLF genes/transcripts. Data normalization and processing are described on the Tabula Sapiens consortium website (https://cellxgene.cziscience.com/) and corresponding publications (62, 63). Dot plots indicate values across the dimensions of color and size of the dots. Dot color approximates gene expression across all cells of that given cell type, while dot size represents the percentage of cells within that cell type expressing the gene (see the scale bar). Cell ontologies: uterine smooth muscle cells (CL:0002601); vascular-associated smooth muscle cells (CL:0000359); T cells (CL:0000084); CD4-positive, alpha, beta T cells (CL:0000624); mature NK T cells (CL:0000814); CD8-positive, alpha, beta T cells (CL:0000625); granulocytes (CL:0000094); neutrophils (CL:0000775); and macrophages (CL:0000235) (https://cellxgene.cziscience.com/cellguide).

To address the import of differential SP/KLF gene expression and thereby function in the uterine myometrium, the relative RNA levels of genes encoding SP/KLF family members were assessed in distinct major cell types found in uterine myometrium, namely uterine smooth muscle cells, vascular-associated smooth muscle cells, and immune cells. The single-cell RNA sequencing (scRNA-seq) database (Tabula Sapiens consortium; https://cellxgene.cziscience.com/) from human non-pregnant uterus (ages 38 and 45 years) was used as a platform for the analyses because of the current lack of accessible scRNA-seq data for term myometrium. As shown in Fig. 2, uterine smooth muscle cells express relatively high amounts of KLF6 > KLF10 > KLF9 transcripts relative to the rest of the KLF members. SPs 1–5 transcript levels were lower and comparable to those of KLFs 5, 7, 8, and 11–17, while SP6–9 transcript levels were undetectable. For uterine vascular-associated smooth muscle cells, transcript levels for KLF9 > KLF6 > KLF2 were predominant, with the rest of the KLFs showing significantly lower or undetectable expression. Transcript levels for SP family members were largely undetectable. Analysis of the same database highlighted a potentially central immunomodulatory role of KLF6 in the uterine microenvironment, given its highest expression in all immune cell types listed relative to the other KLFs (Fig. 2). Interestingly, KLF2, KLF7, KLF10, and KLF12 genes demonstrated expression patterns that were more cell-type variable than KLF6. These collective data suggest functional contributions of specific KLFs to smooth muscle, vascular smooth muscle, and immune gene regulatory networks in the human uterus, while SP family members may have less importance.

Within the complex interplay of different uterine cell types and molecular pathways (Fig. 2), KLF6 may be considered a key player in orchestrating myometrial contractility, given its predominant expression in distinct cell types in the myometrium. However, myometrial expression of KLF6, while highest among all KLFs at term, did not differ in women at term/in labor vs at term/not in labor (Fig. 1). High KLF6 expression has been documented in stromal cells obtained from ectopic women with endometriosis, where it was shown to be anti-proliferative, anti-migratory, and pro-apoptotic (30). While it is not possible to explicitly assign a function for KLF6 in parturition based on the limited information currently available in the literature, it is possible that its high level of expression at term may reflect a regulatory role in the adaptation of smooth muscle and immune cells to oxidative and inflammatory stresses of parturition. In this regard, myometrial hypoxia during labor is central to increased intensity of myometrial contractions (31), and KLF6 expression is up-regulated in a HIF-1a-dependent manner under hypoxic conditions (32).

Another KLF family member of potential importance to term labor is KLF2, given its increased expression in term/in labor vs term/not in labor myometrium, and its consistent expression in various cell types associated with uterine myometrium (Figs 1 and 2). KLF2 actions in the pregnancy myometrium, specifically at parturition and labor onset, have not been reported. However, KLF2 has important regulatory roles in immune cell function during inflammation, in part through its functional interactions and cross-regulation with NFκB (33). In this regard, KLF2 actions in myometrium are perhaps localized primarily to immune cells and inflammation networks, some of which initiate from myometrial smooth muscle cells during parturition.

### SP/KLFs and parturition-associated myometrial gene expression

Significant changes in myometrial gene expression are critical for the transformation of the quiescent myometrium to a highly contractile state for labor onset. Recently published comprehensive reviews on the transcriptional programs associated with labor at term are excellent sources for details on this transformative process (3, 34). Since inflammation and acquisition of the myometrial contractility phenotype are requisite for labor onset, the possible contribution of SP/KLFs to these molecular events is discussed here.

Topologically associated domains (TADs) within nuclear chromatin confer a higher-order level of gene regulation by favoring promoter-enhancer interactions across large expanses of DNA. TADs in the human myometrium have been linked to the transition to labor (16). Significantly, KLF4 has been linked with the ‘open’ TADs of the myometrium of women in labor (16). While little is known about KLF4 gene targets in the myometrium throughout pregnancy, KLF4 is likely implicated in activation of immune/inflammatory gene expression associated with labor onset, consistent with its demonstrated cooperative interactions with the glucocorticoid receptor (GR) in genomic regulatory regions of anti-inflammatory genes in keratinocytes (35), and its well-described role in various inflammatory disorders in other organ systems (36).

KLF5 gene expression has been observed in human term myometrium, with immunohistochemistry revealing that smooth muscle cells stain strongly positive in both nuclear and cytoplasmic compartments (37). Nuclear KLF5 protein abundance was greater in laboring than in non-laboring (preterm Caesarean section) myometrium. Moreover, KLF5 worked in synergy with NFκB at the transcriptional level to induce synthesis and secretion of interleukin (IL)-6 and IL8 in primary cultures of myometrial cells under a pro-inflammatory state mimicking labor (37). The interactivity of NFκB with specific KLFs in chromatin presents an important area for further inquiry.

The KLF9 gene and protein are highly expressed in uterine smooth muscle cells (and nuclei) of pregnant mice (38, 39). Importantly, mice null for KLF9 exhibit delayed parturition by 1–2 days, relative to wild-type counterparts; this was accompanied by reduced myometrial PGR-A levels and reduced myometrial expression of CAP, OXTR, and GJA1 genes at late pregnancy, in the absence of changes in circulating levels of E_2_ and P_4_ (39). In addition, reduced binding in myometrial nuclear extracts to an NFκB binding element, likely reflecting reduced NFκB signaling pathway activity, was noted in the absence of KLF9 expression (39). In women with term (>37 to ≤41 weeks) vs late-term (>41 weeks) pregnancies undergoing Caesarean delivery, myometrial expression of KLF9, total PGR, and PGR-A/PR-B isoform ratio were lower at late-term, underscoring the participation of KLF9 in concert with the PGR-A isoform in the timing of human parturition (40). The accompanying changes (up or down) in transcript levels of select chemokines and cytokines associated with parturition implied transcriptional activator as well as repressor functions for KLF9. Interestingly, expression of contractility-associated (OXTR and GJA1) and clock-related (BMAL, CRYI, PER1, and PER2) genes were not affected by reduced myometrial KLF9 expression, perhaps suggesting a preferential role for KLF9 in orchestrating inflammatory networks rather than myometrial contractility in parturition.

Myometrial contractions during labor are promoted by oxidative stress mediated in part by recruited macrophage release of reactive oxygen species (ROS) (41). Indeed, targeting macrophage-induced ROS production in the myometrium by the antioxidant glutathione prevented preterm birth in a mouse model (42). Consistent with the noted high-level expression of KLF6 in uterine macrophages and uterine smooth muscle cells (Fig. 2) and increased KLF6 expression under conditions of oxidative stress (32), recent chromatin accessibility profiling by ATAC sequencing identified SP/KLF family members and CAP (connexin 43) transcription factor AP-1 in ROS-responsive transcriptional programming (43). While the latter study was conducted in the hormone-responsive MCF-7 breast cancer cell line, results provide support to the notion of SP/KLF family members as ROS-responsive regulators at the level of the chromatin in the myometrium.

### SP/KLFs and steroid hormone signaling

P_4_/PGR action (mediated by PGR-B) in the myometrium during pregnancy is critical for pregnancy maintenance through its suppression of CAP and pro-inflammatory gene expression. Increased PGR-A/PGR-B ratio at term results in induction of myometrial contractility and a pro-inflammatory cascade, leading to initiation of parturition. Thus, nuclear co-regulators that interact with PGR-A or PGR-B directly or indirectly can disrupt the delicate balance in PGR function. Studies described below focus on relevant SP/KLF family members.

KLF9 is a known co-regulator of PRG-B transactivity in uterine endometrial cells (28, 44); thus, it may exert a similar function, albeit with a different PGR isoform (PGR-A) in the myometrium to initiate parturition. In human myometrial cells pre-treated with E_2_ and P_4_ *in vitro*, followed by RU486 to mimic progesterone withdrawal at term, knockdown of KLF9 (by siRNA targeting) led to enhanced PGR-B and ESR1 expression and, concomitantly, to reduced PGR-A isoform and pro-inflammatory CCL3, CXCL1, CXCL5, IL6, and IL11 gene expression (40). These findings highlight KLF9 regulation of myometrial P_4_ sensitivity by altering the PGR-A/PGR-B ratio required for labor onset. In this regard, P_4_ resistance is also a hallmark of endometriosis, and reduced/loss of KLF9 expression in uterine endometrial cells in KLF9 null mice, resulting in increased ectopic lesion establishment (45), and in women with endometriosis (46), was associated with loss of PGR-B expression (by contrast to PGR-A in myometrium) and deregulated expression of a subset of PGR-B target genes. Whether KLF9 directly regulates myometrial PGR-A (increase) or PGR-B (decrease) isoform expression, transactivity, or both for labor onset is presently unclear. In human endometrial stromal cells *in vitro*, co-recruitment of KLF9 and PGR-B to the proximal promoter region of the PGR target gene Dickkopf Wnt signaling pathway inhibitor 1 (DKK1) resulted in their regulation of each other’s transactivity, suggesting that KLF9 provides specificity to and/or fine-tunes the DKK1 promoter response to ligand-dependent PGR signaling (46). Interestingly, in this same study, the gene networks individually regulated by KLF9 and PGR-B are distinct from each other and from those co-regulated by KLF9/PGR-B, with the latter co-regulation showing the most dramatic effects on interferon, chemokine, and cytokine pathways. By extension of these findings to myometrium at labor, it is possible that co-recruitment of KLF9/PGR-A to promoter regions of pro-inflammatory genes may be essential for initiation of parturition, although no report to date has documented their cooperative transactivities at the level of specific immune target genes. It is also possible that KLF9, by increasing PGR-A isoform expression, may enhance NFκB/PGR-A isoform interactions, based on an earlier report of reduced NFκB signaling pathway activity in the absence of KLF9 expression (39) and the well-recognized role of NFκB in functional progesterone withdrawal (47).

The hormone relaxin (RLN) is an important modifier of myometrial phenotype during pregnancy. RLN binds to its receptor RXFP1 in the myometrium to substantially affect (up or down) expression of multiple genes, many of which are also under E_2_ regulation (48). ChIP-seq analyses of these E_2_-regulated genes demonstrate ESR1 binding at the level of the chromatin and predominantly at enhancer regions (48). In an earlier study, RXFP1 was reported as one of the most highly induced genes after co-knockdown (but not individual knockdowns) of PGR and KLF9 in uterine endometrial cells (46). Given that PGR and KLF9 expression in term myometrium is essential for labor onset, and the ability of KLF9 to act as a negative regulator of ligand-dependent ESR1 signaling (e.g., Ishikawa endometrial carcinoma cells) (49), KLF9 and related family members KLF4 and KLF13 may serve to link PGR- and ESR1-mediated RLN/RXP1 actions in the peri-parturient myometrium.

### Hormonal control of SP/KLFs expression

**Figure 3:**
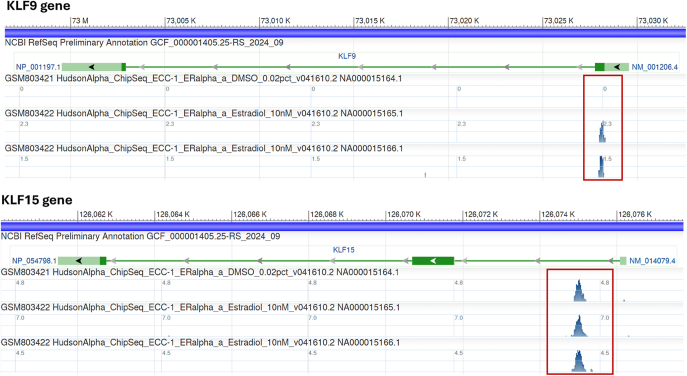
Estrogen receptor α (ESR1) binding to KLF9 and KLF15 genes in the human endometrial epithelial cell line, ECC-1. Upper panel: ChIP-seq tracks for the KLF9 gene were obtained using an antibody to human ESR1. Ishikawa cells were treated with DMSO (vehicle, 0.02%; upper track) or 10 nM estradiol (second and third tracks, replicates) for 1 h, followed by processing for ChIP-seq. Lower panel: same layout as for the upper panel, except that results are for the KLF15 gene. KLF genes are shown in green, with direction of transcription indicated by arrows. Red boxes delineate the region of association of ESR1 with each of the two genes. Designations to the left of each track identify the ENCODE dataset that was queried using the NCBI genome data viewer (https://www.ncbi.nlm.nih.gov/geo/encode/).

Estrogen, progesterone, and glucocorticoids have major roles in regulating pregnancy and parturition; thus, demonstration that SP/KLF family members are under transcriptional control by any of these hormones would further underscore their relevance to parturition. Figure 3 illustrates the association of estrogen receptor α (ESR1) with the KLF9 and KLF15 gene promoters, using data from a ChIP-seq analysis of the human endometrial epithelial cell line ECC-1 (https://www.ncbi.nlm.nih.gov/gdv/browser/geo/?id=GSM1010747%2CGSM803541%2CGSM803422%2CGSM803374). Results demonstrate ligand-dependent ESR1 binding to the KLF9 gene promoter, in contrast to that found for the KLF15 gene, which binds ESR1 in a ligand-independent manner. A comparable analysis of another ECC-1 ChIP-seq dataset (https://www.ncbi.nlm.nih.gov/gdv/browser/geo/?id=GSM803464%2CGSM803340) revealed GR binding to KLF6, KLF9, KLF10, and KLF15 chromosomal genes at promoter/enhancer regions (data not shown). Systematic examination of each KLF gene using a third ChIP-seq database (https://remap2022.univ-amu.fr/) showed PGR binding to KLF2, 6, 7, 9–12, and 15 genes in both term pregnant and non-pregnant myometrium. An exception was the KLF8 gene, where PGR binding was found only in term pregnant myometrium (data not shown). These collective findings provide support for steroid hormone regulation of expression of multiple KLF family members, although the temporal and functional significance of these observations in term pregnancy awaits further inquiry.

Two concepts are worth noting from the above findings. One is the conceivable plasticity of the KLF9 gene in the context of the steroid hormone environment, where its ability to bind ESR1, PGR, and GR might modify its expression and/or transactivity. In previous studies on female reproductive tissues (ovary, endometrium, myometrium), no major changes in expression levels of KLF9 transcript and protein were apparent under normal physiological conditions. However, in pathological states such as delayed labor (40), endometriosis (46), and endometrial carcinoma (50), KLF9 transcript levels (and corresponding protein) were reduced in relevant pathological tissues (myometrium, endometrium, endometrial tumors). These findings may reflect the concomitant reduction of PGR (and/or corresponding increase in ESR1) expression under diseased states (40, 46) and/or aberrancies in PGR interactions with co-regulators, leading to disruptions in their transactivities. The second concept, given the KLF9 gene’s ability to bind both PGR and ESR1, is the possible cross-regulation of KLF9/PGR and KLF9/ESR1 gene expression and the consequences to the antagonistic relationship between ESR1 and PGR actions. The findings that loss of KLF9 expression results in down-regulation of PGR-B and up-regulation of PGR-A, ESR1, and ESR2 gene expression (40, 45, 46, 50), albeit not directly in the context of myometrium at parturition, raise the possibility that KLF9 may skew the normal balance of ESR and PGR isoform expression, thereby leading to pathology. In this regard, KLF9 binding sites in ESR1, ESR2, and PGR gene promoter, enhancer, and/or intronic regions are apparent from analyses of multiple ChIP-seq datasets (data not shown).

**Figure 4:**
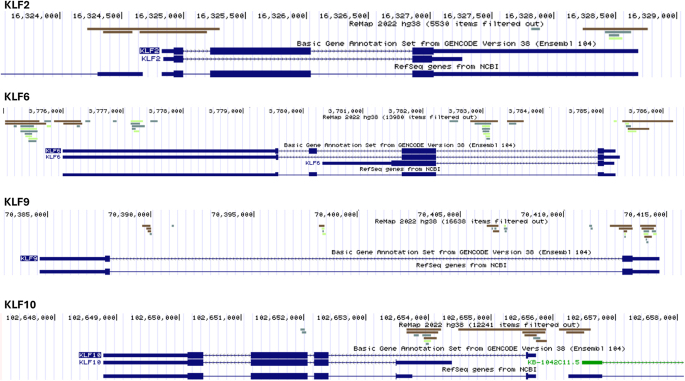
Co-localization of JUN, FOS, and PGR to promoter-enhancer and downstream regions of the major myometrium-expressed KLF genes. ChIP-seq data in the ReMap 2022 database and accessible via the corresponding web portal (https://remap.univ-amu.fr/) were queried for human myometrium ChIP-seq data corresponding to FOS, JUN, and/or PGR binding. The bars (light green for JUN, dark green for FOS, and brown for PGR) indicate regions of binding of each factor to each of the shown KLF genes. The UCSC genome browser was used to generate the tracks shown. The transcript upstream of the KLF2 gene and of opposite transcriptional direction is only partially shown and is an unrelated long noncoding RNA (lncRNA). The transcript upstream of the KLF10 gene and of opposite transcriptional direction (KB-1042C11.5) also is an unrelated lncRNA.

We examined four of the most highly expressed KLF genes (KLF2, KLF6, KLF9, and KLF10) in human myometrium for binding of PGR and activator protein-1 (query: PGR, FOS, and JUN ChIP-seq datasets in the ReMap2022 database/portal; https://remap.univ-amu.fr/hsap_tracks_page) (Fig. 4). Similar to PGR, AP-1 occupancy at gene promoters/enhancers in human myometrium is important to uterine smooth muscle gene regulatory programs in pregnancy. Notably, all four KLF genes exhibited regions of overlap of PGR and AP-1 binding near to or at the corresponding transcription start sites. These same regions also bear the hallmark of acetylation of the histone H3 tail at lysine 27 (H3K27ac), indicative of active transcription at gene enhancers and promoters (data not shown). In the case of the KLF2 and KLF6 genes, we also observed regions of overlap of PGR and AP-1 near or at the 3′ end of the respective KLF chromosomal gene (Fig. 4). These data reinforce the premise that the major human myometrium-expressed KLFs are under control by two of the major myometrial transcription factors (PGR, AP-1), as well as chromatin conformation (H3K27ac mark), all important to myometrial transcriptional activity during pregnancy.

Transforming growth factor β (TGF-β) and thyroid hormone are non-steroidal hormones considered to play significant roles in myometrial transformation for parturition at term (51, 52). Recent studies have described several KLF family members with links to these hormones’ signaling pathways. One study reported that KLF10 and KLF11 are transcriptionally regulated by members of the TGF-β superfamily and, through their pro-apoptotic and anti-proliferative functions, also serve as mediators of TGF-β crosstalk with other signaling pathways (53). Although no direct demonstration has yet been described for parturition events, loss of KLF10 or KLF11 genes resulted in inflammation and immune cell infiltration, hallmarks of myometrium at labor onset, in mouse uterine endometriotic lesions (54). The KLF9 gene is rapidly and robustly induced by thyroid hormone in many cell types and tissues (29). Nevertheless, the relationship of thyroid hormone signaling with KLF9 in myometrium requires further study, based on contrasting reports that levothyroxine administration lowered the risk of pre-term pregnancy in women with subclinical hypothyroidism or thyroid autoimmunity (55), while chronic levothyroxine and acute triiodothyronine treatments enhanced spontaneous uterine contractile activities in human myometrial cells *in vitro* (56).

## Summary and perspectives

The complexity of human parturition and the necessity of its exquisite timing for optimal fetal health underscore the need for a more detailed understanding of the unique roles and impact of participating molecular entities within the biological context of P_4_ and E_2_ signaling networks in the myometrium. In this review, we summarized the current published literature and present our interrogation of publicly accessible databases to support the involvement of SP/KLF family members in regulating myometrial transformative events in parturition. Based on these available data, several family members, by virtue of their myometrial expression at labor, steroid hormone responsiveness, and mediation of steroid hormone signaling networks, are implicated as drivers of parturition. KLFs, by contrast to SP family members, may play more substantial roles in the myometrium at parturition. Among KLFs, KLF9 and KLF6 may contribute to a spectrum of functions preferential to pro-inflammatory pathways rather than to myometrial contractility. Most of these observations, however, are limited in their physiological association to laboring human myometrium at term and are extensions of studies from other non-myometrial targets. Mechanistically defining key KLF target genes requisite to myometrial transformative processes will provide clues into how KLFs may integrate inflammation and myometrial contractility to program key events in parturition.

It is worth noting that while KLF9 and KLF6 genes are robustly expressed in distinct uterine myometrial cell types, their seemingly ubiquitous expression in ‘not in labor’ and ‘in labor’ term myometrium is counterintuitive to physiological significance. A rationale for the apparent discrepancy in levels of expression and anticipated function may be related to the degree of ‘productive’ interactions of these KLFs with PGR-A vs PGR-B isoforms, ESR1, NFκB, and/or AP-1, all of which are subject to post-translational modifications (acetylation, phosphorylation), which can markedly impact their functionality (57). The dissection of the identity, distribution, and crosstalk of functional complexes formed between KLFs and these well-recognized drivers of parturition (PGR, ESR, and AP-1) may provide insights into their relative contributions to normal and pathologic physiology of labor onset.

**Figure 5:**
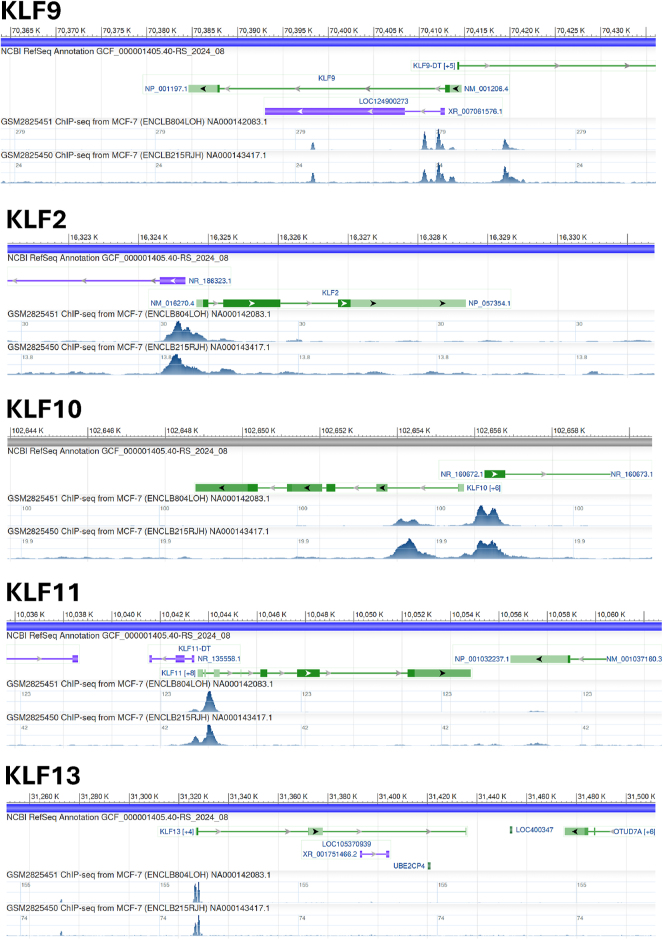
KLF9 binding to proximal upstream regions and introns of chromosomal genes encoding KLF9, KLF2, KLF10, KLF11, and KLF13. Shown are ChIP-seq tracks from human MCF-7 cells stably expressing a C-terminal eGFP-KLF9 fusion protein. Pull-down was with antibody to eGFP-KLF9. GSM2825451, GSM2825450: biological replicates. Data were accessed using the NCBI genome data viewer (https://www.ncbi.nlm.nih.gov/geo/encode/).

Several studies have now demonstrated compensatory, additive, or antagonistic interactions among SP/KLF family members for a given target gene (29). In uterine endometrial cells, KLF9 and KLF13 demonstrate partial overlapping and compensatory functions. Moreover, loss of KLF9 expression results in concomitant loss of KLF4 expression in uterine endometrial carcinoma cells, suggesting KLF4 as a downstream target of KLF9 (50). ChIP-seq analysis of MCF-7 breast cancer cells (https://www.ncbi.nlm.nih.gov/geo/encode/) show that KLF9 and KLF10 are capable of binding to the same region of the KLF2 gene, implicating co-regulation, which may be additive, synergistic, or antagonistic (data not shown). Indeed, parallel ChIP-seq analysis of this same cell line demonstrated that KLF9 associates with its own gene (potential for autoregulation), as well as KLF2, KLF10, KLF11, and KLF13 genes in chromatin (Fig. 5). These potential regulatory networks should be explored in myometrial smooth muscle cells using appropriate ChIP-seq samples to provide important insights into the relative hierarchy of key KLFs in parturition.

It may be important to define new interacting proteins for KLFs, other than those currently known, should their strong potential to serve as parturition ‘drivers’ be further verified. Two candidate proteins are worth noting here and could be targets for future inquiries. Transcription factor 23 (TCF23) is a P_4_-induced target gene, and TCF23 KO mice have a parturition-related phenotype strikingly similar to that of KLF9 KO mice, specifically subfertility along with delayed parturition (58). RNA-seq analyses of myometrium from TCF23 KO mice revealed dysregulated extracellular matrix formation and cell adhesion (58), as was previously noted in human endometrial cells after alterations in steady-state levels of KLF9 (29). The TCF23 protein seemingly lacks DNA-binding activity; therefore, its normal physiology may involve functional interactions with other nuclear and chromatin-associated proteins. The apparent phenocopying of null mutations in KLF9 and TCF23 may indicate their functional interactions with ramifications for parturition. A second candidate protein worthy of further inquiry is T-box transcription factor 2 (TBX2), whose overexpression in human myometrial cells resulted in the suppression of TNFα- and interferon-signaling pathways, as manifested by reduced expression of CXCL10, CXCL11, ISG15, and USP18 genes, among other genes (59). The expression of many of these listed genes was similarly repressed in uterine endometrial stromal and colon cancer cells by KLF9 (29). Thus, we speculate that KLF9 may work in concert with TBX2 in regulating immune signaling by myometrial smooth muscle cells during the transition to labor.

Finally, if specific KLF family members are proven to be drivers of parturition, a valid subsequent question would be whether manipulation of their expression levels may be therapeutically relevant. Recent studies have shown that steroid receptor coactivators, which historically have been considered refractory to manipulation of activity and, hence, function (‘undruggable’), have demonstrated some success as pharmacological targets in cancers (60). While targeting KLF family member expression is not considered a short-term goal for management of labor dysfunctions, fundamental insights into SP/KLF biology and their functional mechanisms at parturition may constitute key initial steps toward this promising therapeutic advance.

## Declaration of interest

The authors declare that there is no conflict of interest that could be perceived as prejudicing the impartiality of this work.

## Funding

Work cited herein from the authors’ research groups was supported by the US National Institutes of Health (NICHD grant number HD21961).
